# Recent Advances in Molecular Diagnostics of Fungal Plant Pathogens: A Mini Review

**DOI:** 10.3389/fcimb.2020.600234

**Published:** 2021-01-11

**Authors:** Ganeshamoorthy Hariharan, Kandeeparoopan Prasannath

**Affiliations:** Department of Agricultural Biology, Faculty of Agriculture, Eastern University, Chenkalady, Sri Lanka

**Keywords:** polymerase chain reaction, molecular identification, next-generation sequencing, fungal plant diseases, emerging fungal pathogens

## Abstract

Phytopathogenic fungal species can cause enormous losses in quantity and quality of crop yields and this is a major economic issue in the global agricultural sector. Precise and rapid detection and identification of plant infecting fungi are essential to facilitate effective management of disease. DNA-based methods have become popular methods for accurate plant disease diagnostics. Recent developments in standard and variant polymerase chain reaction (PCR) assays including nested, multiplex, quantitative, bio and magnetic-capture hybridization PCR techniques, post and isothermal amplification methods, DNA and RNA based probe development, and next-generation sequencing provide novel tools in molecular diagnostics in fungal detection and differentiation fields. These molecular based detection techniques are effective in detecting symptomatic and asymptomatic diseases of both culturable and unculturable fungal pathogens in sole and co-infections. Even though the molecular diagnostic approaches have expanded substantially in the recent past, there is a long way to go in the development and application of molecular diagnostics in plant diseases. Molecular techniques used in plant disease diagnostics need to be more reliable, faster, and easier than conventional methods. Now the challenges are with scientists to develop practical techniques to be used for molecular diagnostics of plant diseases. Recent advancement in the improvement and application of molecular methods for diagnosing the widespread and emerging plant pathogenic fungi are discussed in this review.

## Introduction

Fungal plant pathogens are among the foremost biotic factors that cause devastating disease in crops (Doehlemann et al., 2017). About 8,000 species of fungi and oomycetes are linked with diseases in plants (Horst, 2008; Fisher et al., 2020). Pathogenic fungi infect plants at any phase from the seedling stage to the seed maturing stage under natural environmental conditions, either alone or in concert with other kinds of phytopathogens (Narayanasamy, 2011). The most common diseases caused by plant pathogenic fungi are anthracnose, blight, canker, damping off, dieback, gall, leaf spot, powdery mildew, rust, root rot, scab, and wilt (Iqbal et al., 2018; Hussain and Usman, 2019; Jain et al., 2019). These diseases can generate significant losses in yield (Godfray et al., 2016), quality and quantity (Shuping and Eloff, 2017) in various agricultural systems (Rodriguez-Moreno et al., 2018) of economically important agronomical (Leonard and Szabo, 2005; Asibi et al., 2019), horticultural (Agrios, 2009; Wenneker and Thomma, 2020), floricultural and ornamental (Darras, 2016; Lecomte et al., 2016), and forest (Ritz, 2005; Marčiulynas et al., 2020) plant species worldwide (Malcolm et al., 2013).

The increasing world population necessitates well-organized plant disease management and control in agriculture to assure food security and safety (FAO et al., 2018; Sarrocco and Vannacci, 2018). An efficient and effective framework for early alert and quick response is a crucial element to combat against phytopathogenic fungi (Sankarana et al., 2010; Nagrale et al., 2016). Diagnosis of fungal plant pathogen is of significance in the area of plant protection as it contributes to improving crop vigor and health. Therefore, fungal disease management requires accurate diagnosis of diseases which is chiefly based on the identification of causative agents. Moreover, it is essential to confirm fungal plant diseases even though the diagnosis of such diseases based on the external symptoms is already made to a satisfactory level. Further, an entire list that covers a known plant disease, its typical sign and symptoms, and its known potential phytopathogen for a precise host is a requisite for disease diagnosis (Thind, 2015).

Various advances have been made in the field of phytopathogenic fungal diagnosis. Conventional fungal disease diagnostic methods have utilized visible signs after phytofungal infections including propagules of fungi *viz.* conidia, sclerotia, or mycelia on the external surfaces of flora, or fungal disease symptoms caused by fungal pathogens after infection (Nezhad, 2014; Tör and Woods-Tör, 2017). These approaches are the cornerstone of fungal disease diagnostics. Widely used conventional methods include isolation and culturing, reinoculation, microscopic techniques and biochemical tests (Tan et al., 2008; Sharma and Sharma, 2016), which have some drawbacks in that they are tedious and require knowledge and expertise in fungal plant pathology and taxonomy (McCartney et al., 2003; Pryce et al., 2003). Immunological-based diagnostic methods are built on the antigen-antibody binding principle and some issues have been noted, such as low sensitivity and affinity in assays, and potential interference from contaminants (Meng and Doyle, 2002). Furthermore, detection of fungal plant pathogens has not been effective due to the high inconsistency and phenotypic serological plasticity of fungi (Luchi et al., 2020). Thus, the implementation and development of novel and effective diagnostic methods to thwart fungal plant disease are urgent. For these reasons, plant-fungal diagnosis has moved to molecular approaches that facilitate pathogen recognition and quantification. Molecular assays can overcome drawbacks of conventional and serological methods in fungal diagnostics.

Modern developments use high throughput molecular detection strategies for plant infecting fungi. These include standard polymerase chain reaction (PCR), real-time PCR, nested PCR, loop-mediated isothermal amplification (LAMP), rolling circle amplification (RCA), and nucleic acid sequence-based amplification (NASBA) (Aslam et al., 2017; Cheng et al., 2020). PCR restriction fragment length polymorphism (PCR-RFLP) and PCR denaturing-gradient gel electrophoresis (PCR-DGGE) are the methods suitable for genotyping more than for species identification (Johnston‐Monje and Mejia, 2020). Further, molecular techniques cover magnetic capture- hybridization PCR (MCH-PCR), *in situ* PCR, co-operational PCR, multiplex PCR, DNA macro and micro arrays, next-generation sequencing (especially RNA-Seq based), etc. (Kumar et al., 2016). Greater confidence, accuracy, specificity, and sensitivity of DNA based molecular techniques (Capote et al., 2012; Midorikawa et al., 2018) permit the diagnosis of phytopathogens at primary stages of infection even though they are present at lower DNA concentrations (Luchi et al., 2013; Rollins et al., 2016).

Additionally, bioinformatics databases such as GenBank at the National Centre for Biotechnology Information (NCBI), Nucleotide Sequence Database Collaboration at the European Bioinformatics Institute (EBI), MycoBank, etc. offer platforms for documenting mycological nomenclatural novelties, storing and retrieving facilities of nucleotide sequences of plant infecting fungi which further accelerate the molecular tools to potentially diagnose and perform species delimitation among existing and evolving fungal species. Rapidly emerging and novel fungal plant pathogens threaten the global economy. Hence, rapid and accurate detection and identification of phytopathogenic fungi is crucial. This review aims to summarize various molecular techniques in fungal plant pathogen diagnosis along with their advantages and drawbacks. It also examines the available molecular tools used to diagnose previously present, emerging, and re-emerging plant pathogenic fungi in various agricultural crops.

## Molecular Tools for Detection of Fungi

### Preanalytical Steps in Molecular-Based Diagnosis of Plant-Infecting Fungi

Detection and diagnosis of fungal plant pathogens using molecular techniques require preanalytical steps such as genomic DNA extraction that efficiently lyse fungal cells and recover the DNA, purification and quantification of extracted DNA. Several protocols for isolation of DNA from plant infecting fungi are available (Doyle, 1991; Cenis, 1992; Chi et al., 2009; Zhang et al., 2010; Gontia-Mishra et al., 2014; Yang et al., 2016). Recent fungal plant disease diagnostic approaches use commercial DNA extraction kits (Martinelli et al., 2015; Moffat et al., 2015). However, laboratories are dependent on standard protocols embracing lyophilization of mycelia, disruption of chitin cell wall by grinding and isolation of DNA in a buffer containing chemicals, removal of proteins by phenol-chloroform mixture, and precipitation with propanol. The isolated fungal DNA can then be purified using standard methods including pelleting, silica membrane (Mancini et al., 2016), spin filter, and silica coated magnetic particle separation (Tsui et al., 2011). Finally, the concentration of fungal DNA in the samples can be determined using UV spectrophotometer and further it can be diluted with ultrapure PCR grade water to provide an appropriate DNA concentration (Abdullah et al., 2018).

### Polymerase Chain Reaction (PCR) Based Assays

#### End-Point PCR

Advent of PCR revolutionized the accurate identification of various plant pathogens in disease management, including fungi (Ma and Michailides, 2007). In this *in vitro* technique, a piece of DNA template is exponentially amplified (Caetano-Anolles, 2013) through repeated cycles of denaturation, annealing, extension, final extension, and final hold reactions at various temperatures using specific primers, deoxyribonucleotide triphosphates (dNTPs), and a thermostable Taq DNA polymerase in buffer solution (Griffiths, 2014). In end-point PCR, designing either specific oligonucleotides that target certain fungal species or universal primers to amplify multiple pathogens followed by sequencing allows the accurate detection of fungal plant pathogens. For each set of nucleotide sequences of fungal isolates, the identity of each isolate can be determined by comparison against ex-type cultures available in the NCBI GenBank database using Basic Local Alignment Search Tool (BLAST) analysis. The presence of a target revealed in agarose gel electrophoresis assures the existence of targeted phytopathogenic fungi (Mirmajlessi et al., 2015).

End-point PCR systems are considered a cost-effective choice compared to other existing molecular diagnosis options for fungal plant pathogens. However, end-point PCR assays can be time-consuming and it is difficult to design primer sets to delineate closely-related fungal pathogens. Sikdar et al. (2014) dealt with diagnosing *Phacidiopycnis washingtonensis* and *Sphaeropsis pyriputrescens* (which cause speck rot and *Sphaeropsis* rot diseases in apple, respectively) using end-point PCR and real-time PCR assays. They found that the quantitative real-time PCR approach was more sensitive than the end-point PCR approach for rapid diagnosis. Different types of PCR-based molecular diagnosis and examples of each type are given in **Table 1**.

**Table 1:** PCR based approaches to diagnose pathogenic fungi in different crops.

Assay	Diagnosed fungi	Host	Disease	Target gene^a^	Reference
End-point PCR	*Cercospora tezpurensis* sp. nov.	*Capsicum assamicum*	Leaf spot	*ACT*, *CAL*, *HIS* and *TEF-1α*	Meghvansi et al., 2013
End-point PCR	*Exobasidium maculosum*	Blueberry	Leaf and fruit spot	*LSU-rDNA*	Brewer et al., 2014
End-point PCR	*Golovinomyces cichoracearum sensu lato*	*Cannabis sativa*	Hemp powdery mildew	*ITS*	Pépin et al., 2018
End-point PCR	*Cercospora* cf*. flagellaris*	*Cannabis sativa*	Hemp leaf spot	*ITS, TEF-1α, CAL, HIS* and *ACT*	Doyle et al., 2019
End-point PCR	*Neopestalotiopsis clavispora* and *Colletotrichum siamense*	Macadamia	Leaf spot	*ITS, TUB2, TEF-1α*, *ACT* and *GAPDH*	Prasannath et al., 2020
Nested PCR	*Puccinia striiformis* f. sp. *tritici*	Wheat	Stripe rust	*PSR*	Wang et al., 2009
Nested PCR	*Phytophthora cactorum *	Strawberry	Crown rot	*ITS*	Bhat and Browne, 2010
Nested PCR	*Colletotrichum gloeosporioides*	*Dioscorea* spp.	Greater yam anthracnose	*ITS*	Raj et al., 2013
Nested PCR	*Pilidiella granati*	Pomegranate	Twig blight and crown rot	*SSU-rDNA*	Yang X. et al., 2017
Multiplex PCR	*Fusarium* *Verticillioides* and* F. subglutinans*	Maize	Stalk rot and ear rot	*gaoB*	Faria et al., 2012
Multiplex PCR	*Neofabraea alba*, *N. perennans* and *N. keinholzii*	Apple	Bull’s eye rot	*TUB2*	Michalecka et al., 2016
Multiplex PCR	*Fusarium oxysporum *f. sp.* cubense *lineage VI strains	*Musa* spp.	Dessert/beer bananas	*TEF-1α* and *RPC2*	Ndayihanzamaso et al., 2020
Quantitative PCR	*Didymella bryoniae*	Cucurbits	Gummy stem blight	*RAPD*	Ling et al., 2010
Quantitative PCR	*Ramularia* *collo-cygni *	Barely	Ramularia leaf spot	Not mentioned	Havis et al., 2014
Quantitative PCR	*Rhizoctonia solani*	Tobacco	Target spot	*ITS*	Zhao Y. Q. et al., 2014
Quantitative PCR	*Magnaporthe oryzae*	Rice	Rice blast	*18S-28S rDNA*	Sun et al., 2015
Quantitative PCR	*Verticillium longisporum *	*Brassica napus*	Wilt and stem stripe	*TUB2*	Depotter et al., 2017
Quantitative PCR	*Pyrenophora tritici-repentis* and *Parastagonospora nodorum*	Wheat	Tan (yellow) spot and Septoria blotch	*ToxA*	Abdulla et al., 2018
Quantitative PCR	*Fusarium culmorum *	Cereals	Foot and root rot and Fusarium head blight	*COX2*	Bilska et al., 2018
Quantitative PCR	*Fusarium guttiforme*	Pineapple	Fusariosis	*TEF-1α* and *TUB2*	Carnielli-Queiroz et al., 2019
End-point PCR and quantitative PCR	*Phacidiopycnis washingtonensis* and *Sphaeropsis pyriputrescens*	Apple	Speck rot and Sphaeropsis rot	*ITS*	Sikdar et al., 2014
End-point PCR and quantitative PCR	*Guignardia citricarpa*	*Citrus* spp.	Citrus black spot	*ITS*	Faganello et al., 2017

^a^ITS, internal transcribed spacer; LSU-rDNA, large subunit ribosomal DNA; SSU-rDNA, small subunit ribosomal DNA; TUB2, β-tubulin 2; TEF-1α, translation elongation factor 1-α; GAPDH, glyceraldehyde-3-phosphate dehydrogenase; ACT, actin; CAL, calmodulin; HIS, histone H3; PSR, Puccinia striiformis f. sp. tritici repeat sequence; gaoB, galactose oxidase B; RPC2, DNA-directed RNA polymerase III subunit RPC2; RAPD, random amplified polymorphic DNA; ToxA, exotoxin A; COX-2, cyclooxygenase-2.

#### Nested PCR

Nested PCR is a modified version of end-point PCR that uses two sets of primer pairs aimed at two rounds of PCR amplification to enhance specificity and sensitivity. Nesting also aids usage of comparatively non‐specific PCR primers in the initial round of PCR for amplification of numerous pathogens, followed by the use of pathogen‐specific primers in the next round (Bhat and Browne, 2010). Twig blight and crown rot of pomegranate are emerging diseases in pomegranate cultivation that are caused by *Pilidiella granati*. A nested PCR assay improved both sensitivity and detection of *P. granati* and made it possible to diagnose the causative agent when the sample contained DNA as low as 10 pg of *P. granati* (Yang X. et al., 2017). Great yam disease caused by *Colletotrichum gloeosporioides* (Raj et al., 2013) and eucalyptus dieback disease caused by *Cylindrocladium scoparium* (Qiao et al., 2016) were also detected by this technique. Sensitivity of detection using nested PCR could be enhanced from 10- to 1000-fold over an end-point PCR assay (Ippolito et al., 2002; Silvar et al., 2005). However, nested PCR assays are time-consuming and have an increased risk of cross-contamination due to the manipulation of previously-amplified samples, which can create false-positive outcomes (Raj et al., 2013). Therefore, nested PCR and end-point PCR methods that may produce amplicon contamination would not be recommended to be used as reliable diagnostic methods.

#### Multiplex PCR

Multiplex PCR assay uses one reaction mixture with various primer pairs, and allows simultaneous amplification of several pathogens (Sint et al., 2012). The generated amplicons can then be separated and visualized using electrophoresis. Designing primers for the multiplex assay is crucial, and specific sets of primers should have similar annealing temperatures for successful amplification (Zhao X et al., 2014). A concurrent diagnostic assay to detect 12 fungi associated with fruit rot in cranberry was established using the multiplex PCR method. Fungal pathogens *Allantophomopsis cytisporea*, *A. lycopodina*, *Phyllosticta elongate*, *Coleophoma empetri*, *Colletotrichum fiorinae*, *C. fructivorum*, *Fusicoccum putrefaciens*, *Monilinia oxycocci*, *Phomopsis vaccinia*, *Phyllosticta vaccinia*, *Physalospora vaccinia*, and *Strasseria geniculate* linked with cranberry fruit rot were effectively identified with the use of *ITS-LSU* and *TEF-1α* gene regions (Conti et al., 2019). The pathogenic fungi *Fusarium oxysporum*, *Bipolaris cactivora*, *Phytophthora nicotinae*, and *Phytophthora cactorum* are threats to the cactus industry that potentially affect its export sector. This problem has been resolved using multiplex PCR assays. It was noted that the diagnosing tool was sufficient to detect and identify these quarantine fungal pathogens in grafted cacti (Cho et al., 2016). Though multiplex PCR assays are quick and reliable in nature, the assays are potentially expensive and resource-intensive, and decreased sensitivity associated with the multiplex methods (Pallás et al., 2018).

#### Quantitative PCR

Quantitative PCR (qPCR), permits detection and quantification of particular DNA or RNA sequences of phytopathogenic fungi in a PCR reaction mixture in real-time. The relative number of copies of target DNA and RNA sequences can be estimated by projecting a *Ct* (cycle threshold) value of the fungal samples using sequence-specific primers (Balodi et al., 2017). Fluorescent dyes such as SYBR Green I, Eva Green, Molecular Beacons, or sequence-specific fluorescence-labeled reporter probes such as TaqMan (Badali and Nabili, 2012) are used to monitor the reaction during amplification steps. The basic principle is that the fluorescent signal is proportional to the amount of amplicon produced in each cycle and can be generated by an intercalating dye or from the breakdown of a dye-labeled reporter probe during amplification (Alemu, 2014). A hypervirulent and emerging fungal plant pathogen, *Cryphonectria parasitica* causes various diseases (blight, lethal bark cankers, wilting, and dieback) in chestnut trees, *Castanea dentata* and *C. sativa* (Murolo et al., 2018; Jain et al., 2019). Molecular diagnosis by qPCR allowed the detection of *C. parasitica* using *rDNA ITS* sequences with a sensitivity of 2 fg of genomic DNA which was equivalent to the single spore of a pathogen (Chandelier et al., 2019).

An emerging fungal pathogen *Ramularia collo-cygni* shows typical symptoms of small, brown spots on leaves, sheaths, and awns, which made it difficult to accurately diagnose this disease using conventional techniques (Havis et al., 2015). A qPCR test was developed and submitted as the first report on molecular detection of *R. collo-cygni* in barley seed (Havis et al., 2014). An aggressive and emerging British *Verticillium longisporum* is another example of a novel fungal pathogen that was diagnosed with a qPCR approach (Depotter et al., 2017). A qPCR was able to distinguish and quantify *Diaporthe helianthi* and *D. gulyae*, the fungal pathogens of Phomopsis stem canker in sunflower. These causative agents from the same genus were successfully screened using the assay (Elverson et al., 2020). *Pyrenophora tritici-repentis* and *Parastagonospora nodorum* cause co-infections in wheat and share common physiognomies, making it a challenge for traditional disease diagnosis. Two dual-labeled probes with unique fluorogenic reporters (permitting DNA sequences from *P. triticirepentis* and *Pa. nodorum* to be amplified in parallel but independently of each other) were custom designed to perform a duplex qPCR assay, and results obtained were accurate and appropriate to simultaneous differentiation and suitable for high throughput screening of multiple pathogens (Abdullah et al., 2018). This technique is fast and very sensitive (Sikdar et al., 2014), and can provide reliable information on pathogen load (Garrido et al., 2009) and high throughput quantification of target DNA in biological areas (Schena et al., 2013). Further, the TaqMan probe offers an extra level of specificity (Shuey et al., 2014). However, qPCR needs a specialized instrument and cost of the instrument and probe can be high (Abdullah et al., 2018).

#### BIO-PCR

BIO-PCR assay is a modification of end-point PCR technique which involves a pre-assay incubation step in a diseased sample to increase the biomass of the causal agent. This technique is mainly used to concentrate target pathogens by growing the target pathogen in a growing media that prevent the growth of non-target microorganisms to improve detection (Schaad et al., 1995) and has been effectively used to detect seed-borne fungal pathogens (Kumar et al., 2020). A seed and airborne lupin anthracnose disease caused by *Colletotrichum lupini* was diagnosed using the BIO-PCR method. Incubation of the seeds with amended Yeast Malt Broth was done to enrich *C. lupine* biomass and a species-specific primer pair was developed based on *rDNA IGS* sequence. The established BIO-PCR protocol allowed the detection of *C. lupine* in *Lupinus* spp. (Pecchia et al., 2019). The seed-borne fungal pathogens *Alternaria alternata*, *A. radicina*, and *A. dauci* were detected using specific primers of *ITS* in rDNA with the help of a deep-freeze-blotter method during the BIO-PCR assay (Konstantinova et al., 2002). High sensitivity, elimination of PCR inhibitors and detection of living cells to avoid false positives are the advantages over end-point PCR techniques (Marcinkowska, 2002; Fatmi et al., 2005). Limitations of this technique are that it is time consuming and costs are incurred when selective media is used for the assay (Schena et al., 2004; Mancini et al., 2016).

#### Magnetic-Capture Hybridization PCR

Magnetic-capture hybridization PCR (MCH-PCR) uses DNA isolation with a purification phase that contains hybridization with single stranded DNA (ssDNA) probe on magnetic beads followed by the PCR amplification of target DNA sequences (Jacobsen, 1995). This PCR assay was chiefly established to deal with PCR inhibitors in plant extracts during DNA isolation steps. The magnetic beads used are coated with a biotinylated oligonucleotide that is specific to a DNA region of the fungal pathogen of interest (Walcott et al., 2004). The hybridization of double stranded DNA (dsDNA) and magnetic beads allows for separation of the complex from inhibitors (Capote et al., 2012). MCH and real-time based PCR assays were evaluated for two cucurbit seed pathogens, *Acidovorax avenae* subsp. *citrulli* and *Didymella bryoniae*, that cause bacterial fruit blotch and gummy stem blight, respectively. The assay facilitated simultaneous detection of both tested pathogens in cucurbit seed samples (Ha et al., 2009). Designing of a capture probe used in MCH-PCR involves selection of oligonucleotide probe sequence from highly conserved regions of fungal pathogens (Langrell and Barbara, 2001). The selected sequence can be examined *in silico* for specificity using BLAST. The 5’ end of the probe is then biotin-labeled (Chen and Griffiths, 2001) to allow attachment with streptavidin-coated magnetic beads (Johnson et al., 2013). MCH-PCR would decrease the total detection time, increase PCR sensitivity, and remove most of the inhibitors of the amplification reaction and excess of nontarget DNA (Amagliani et al., 2006).

### Isothermal Amplification Based Methods

#### Rolling Circle Amplification

Rolling circle amplification (RCA) is an isothermal enzymatic assay that exploits DNA or RNA polymerase to generate ssDNA or RNA molecules. Requirements for an RCA assay are DNA polymerase, homologous buffer, short DNA/RNA primer, a circular template, and deoxynucleotide triphosphates (Gu et al., 2018). In this assay, DNA amplification using phi29 DNA polymerase with a strand displacement activity to extend a single or multiple primers annealing to a circular DNA template is essential (van Emmerik et al., 2020). The strand displacement process allows newly synthesized DNA template to displace the formerly synthesized DNA molecule to release ssDNA (Bhat and Rao, 2020). Long ssDNA with 100– 1,000 tandem repeats of the original targeted sequence are generated after cascade of strand displacement events (Kieser and Budowle, 2020). Four padlock probes PLP-Nm, PLP-Np, PLP-Nk, and PLP-Nv targeted on *TEF-1α* segments were designed to identify *Neofabraea malicorticis*, *N. perennans*, *N. kienholzin*, and *N. vangabunda* that cause bull’s eye rot in apple. An RCA assay was used to diagnose these pathogens and provided effective and sensitive results to monitor the pathogens in the quarantine sector (Lin et al., 2018). In another study, RCA was performed to detect the Fusarium head blight-causing agent *Fusarium graminearum* and other pathovars, *F. oxysporum*, *F. incarnatum-equiseti*, and *F. tricinctum* species complex with the use of padlock probes that were designed based on polymorphisms in the elongation factor *TEF-1α* (Davari et al., 2012). Simplicity, efficiency, and no need of temperature cycling devices are the advantageous of the RCA assay (Dong et al., 2013; Goo and Kim, 2016). This method can also be used to analyze gene expression, single nucleotide polymorphism, mRNA splicing and post translational modification of protein molecules (Gao et al., 2019).

#### Loop Mediated Amplification

Loop mediated amplification (LAMP) technique has become a significant diagnostic tool in various plant disease diagnosis over only a decade and holds huge potential in plant disease management (Le and Vu, 2017). The LAMP reaction comprises two main stages including an initial step and cycling amplification, followed by an elongation step (Panno et al., 2020). In a LAMP assay, a set of two internal primers, forward inner primer (FIP) and backward inner primer (BIP), one backward loop primer (B-Loop), and another set of two outer primers (F3 and B3), are used to recognize six unique sequences on the targeted nucleic acid. Each FIP and BIP comprises double distinct sequences corresponding to sense and anti-sense strands of targeted DNA. Inclusion of two extra loop primers such as loop forward (LF) and loop backward (LB) and Bst DNA polymerase may accelerate the LAMP method (Nagamine et al., 2002; Francois et al., 2011). Its high exponential and isothermal amplification yields 10^9^–10^10^ fold target DNA in 45–60 min at 60–65°C and this temperature rage is considered to be ideal for Bst polymerase activity (Notomi et al., 2000; Chander et al., 2014). Examples of various fungal plant disease diagnoses using LAMP are shown in **Table 2**.

**Table 2:** LAMP based molecular diagnosis of fungal pathogens in various crops.

Pathogen	Host	Disease	Target gene^a^	Detection system	Reference
*Phytophthora sojae*	Soybean	Phytophthora root rot	*A3aPro*	Real time measurement of turbidity, gel electrophoresis, and hydroxynaphthol blue (HNB) visualizing indicator	Dai et al., 2012
*Verticillium dahliae*	Olive	Vascular wilt	*RAPD*	HNB visualizing indicator	Moradi et al., 2014
*Botrytis cinerea*	Fruits and flowers	Gray mold disease	*Bcos5*	HNB visualizing indicator	Duan Y. B. et al., 2014
*Fusarium oxysporum* f. sp. *ciceris *	Chick pea	Fusarium wilt	*TEF-1α*	HNB visualizing indicator	Ghosh et al., 2015
*Colletotrichum falcatum*	Sugarcane	Red rot	*SCAR*	SYBR Green I dye	Chandra et al., 2015
*Didymella bryoniae*	Cucurbitaceae	Gummy stem blight	*RAPD*	Calcein indicator and gel image	Yao et al., 2016
*Plasmopara viticola*	Grape	Grape downy mildew	*ITS*	HNB visualizing indicator	Kong et al., 2016
*Calonectria osedonaviculata* and *C. henricotiae*	Box wood	Boxwood blight	*TUB2*	Capillary gel electrophoresis	Malapi-Wight et al., 2016a
*Fusarium oxysporum* f. sp. *lycopersici*	Tomato	Tomato wilt	*SIX3*	Melting curve analysis	Ayukawa et al., 2017
*Peronospora destructor*	Onion	Downey mildew	*ITS*	HNB visualizing indicator	Yang K. et al., 2017
*Pyrenopeziza brassicae*	*Brassica napus*	Light leaf spot	*ITS* and *TUB2*		King et al., 2018
*Fusarium fujkuroi* and *Magnaporthe oryzae*	Rice	Bakane and rice blast	*TEF-1α* and *CAL*	Gel electrophoresis	Ortega et al., 2018
*Puccinia triticina*	Wheat	Leaf rust	*PTS68*	HNB visualizing indicator	Manjunatha et al., 2018
*Fusarium fujkuroi*	Rice	Bakane	*NRPS31*	HNB visualizing indicator	Zhang et al., 2019
*Magnaporthe oryzae*	Rice	Rice blast	*MGG_04322*	HNB visualizing indicator and gel electrophoresis	Li et al., 2019
*Ustilago tritici*	Wheat	Loose smut	*LSU-rDNA* and *ITS*	Visual observation under natural light and gel imager	Yan et al., 2019
*Fusarium oxysporum* f. sp. *melonis*	Melon	Fusarium wilt	*TEF-1α*	HNB visualizing indicator	Almasi, 2019
*Uromyces betae*	Sugar beet	Sugar beet rust	*cyt b*	FAM dye	Kaczmarek et al., 2019
*Colletotrichum gloeosporioides*	Strawberry	Anthracnose	*cyt b*	HNB visualizing indicator	Wu et al., 2019
*Talaromyces flavus *	Strawberry	Not mentioned	*rlf*	Fluorescence data	Panek and Frąc, 2019
*Alternaria* spp.	Pear	Pear black spot	*Aacyt-b*	SYBR Green I dye	Yang et al., 2019
*Calonectria ilicicola, Dactylonectria macrodidyma* and *Dactylonectria* genus	Avocado	Black root rot	*TUB2* and *HIS*	Presence of annealing curve	Parkinson et al., 2019
*Phytophthora infestans*	Potato	Potato late blight	*PiSMC*	HNB visualizing indicator	Kong et al., 2020

^a^ITS, internal transcribed spacer; LSU-rDNA, large subunit ribosomal DNA; TUB2, β -tubulin 2; TEF-1α, translation elongation factor 1-α; CAL, calmodulin; HIS, histone H3; PTS68, Puccinia triticina sequence repeat; RAPD, random amplified polymorphic DNA; Bcos5, Botrytis cinerea mitogen-activated protein kinase; SIX3, secreted in xylem 3; NRPS31, non-ribosomal peptide synthetase; MGG_04322, A1b1 superfamily hypothetical protein; cyt b, cytochrome b; rlf, DNA replication licensing factor; Aacyt-b, Alternaria alternata cytochrome b; PiSMC, Phytophthora infestans specific multiple copy DNA sequence; SCAR, sequence characterized amplified region; A3aPro, an element in the upstream of the avirulent gene Avr3 in Phytophthora sojae.

*Uromyces betae*, sugar beet rust-causing fungi, was identified within 30 min using LAMP assay which targeted the *cytochrome b* DNA sequence (Kaczmarek et al., 2019). Conventional LAMP (cLAMP) and a quantitative LAMP (qLAMP) assays were performed to diagnose a quarantine fungal pathogen *Fusarium circinatum* that causes pitch canker in pine and other conifers. LAMP probes targeting *TEF-1α* revealed that qLAMP tests had higher specificity than cLAMP for the detection of *F. circinatum* (Stehliková et al., 2020). A widespread pathogen, *Sclerotinia sclerotiorum*, with a broad host range including rape seed, was detected using a LAMP assay targeting *Ssos5* and a visualizing indicator hydroxynaphthol blue (HNB) for detection. The identification limit of *S. sclerotiorum* using the LAMP technique was 0.1 fg µl^−1^ of genomic DNA per reaction which was significantly lower (100 fg μl^−1^) than the end-point PCR test (Duan Y. et al., 2014). Further, the first LAMP and qLAMP detection systems using *MGG_04322* as a target in *Magnaporthe oryzae* were developed for prompt detection and accurate identification of rice blast disease (Li et al., 2019). *Fusarium odoratissimum* tropical race 4 (TR4), the cause of panama disease in banana plants, was accurately diagnosed based on a LAMP assay using *TR4* markers that were sequenced from diversity arrays technology sequencing (DArTseq) technology (Ordóñez et al., 2019). LAMP allows analysis of crude samples as it is not affected by inhibitors (Panno et al., 2019). This tool is extremely sensitive and specific due to amplification of nucleic acids accomplished by using up to six primers (Becherer et al., 2020). Isothermal and energy efficient intensification requirements of LAMP technology makes it a prime candidate for rapid and inexpensive alternative assays (Waliullah et al., 2020). Thus, LAMP is well established in several areas including medicine, agriculture, and food industries (Mori and Notomi, 2009; Guan et al., 2010; Panno et al., 2020). The short size of target gene fragments, using six primers that can generate difficulties in experimental design, and the extreme amount of indicator and other reaction constituents that inhibit polymerase and carryover contaminations are considered to be the drawbacks of this diagnostic tool (Tanner et al., 2015).

#### Nucleic Acid Sequence Based Amplification

Nucleic acid sequence based amplification (NASBA) amplifies nucleic acids under isothermal conditions. It commonly uses RNA for amplification where a single stranded RNA (ssRNA) template is changed to complementary DNA (cDNA) by reverse transcriptase. The temperature for NASBA is around 41°C and the assay utilizes avian byeloblastosis virus reverse transcriptase, RNase H, and T7 RNA polymerase (Chang et al., 2012). It has the ability to generate over 10^8^ copies of the desired nucleic acid sequence within 30 min (Wernecke and Mullen, 2014) and amplicons are detected *via* a probe-capture hybridization using electrochemiluminescence in molecular beacons (Heo et al., 2019). NASBA does not require a thermal cycler and the amplification power is comparable or better than real-time based PCR assays (Loens et al., 2006). Moreover, NASBA requires only short reactions, has high sensitivity and stringent control, and is not affected by inhibitors and is well suited for lab-on-a-chip devices (Honsvall and Robertson, 2017). The application is very rare in plant fungal pathogen detection. However, it has potential to be used in the detection of fungal diseases in future.

### Post Amplification Techniques

#### DNA Microarray

A DNA microarray (DNA chip, gene chip, or biochip) (Bhatia and Dahiya, 2015) is an assemblage of microscopic DNA spots attached to a solid surface (usually glass) in defined positions. The microscopic spots consist of thousands of specific DNA sequences (probes) that are used to hybridize a cDNA (target) sample. Hybridized probe-cDNA systems can be detected and quantified using fluorophore, silver, or chemiluminescence-labeled targets to define the comparative abundance of transcripts in the sample (Guigó, 2013). Advancement of DNA microarray technology has led high throughput and multiple detection of various phytopathogens including viruses, viroids, bacteria, and fungi (Tiberini and Barba, 2012; Musser et al., 2014; Nam et al., 2014; Krawczyk et al., 2017). PCR primers and florescent probes for targeting fungal potato pathogens *Rhizoctonia solani*, *Spongospora subterranea* (*ITS* region), *Alternaria solani*, *A. alternata* (*Alt_a1* gene), *Fusarium* sp. (*TEF-1α*), and *Colletotrichum coccodes* (*TUB2*) were used with qPCR microarray technology in 48-well silicon microarrays (Nikitin et al., 2018). A novel microarray assay (ArrayTube) using marker genes *ITS*, *TEF-1α*, and *16S rDNA* with well performing probes allowed the detection of various sugar beet root rot pathogens, *Aphanomyces cochlioides*, *Botrytis cinerea*, and *Penicillium expansum* (Liebe et al., 2016). DNA microarrays can be produced batch wise on standard microscope slides in a rapid, easy, consistent, and cost-efficient way (Johannes et al., 2020). A major drawback of microarray is that it can only detect sequences that the array is designed to identify (Bumgarner, 2013).

#### DNA Macroarray

DNA macroarrays are built by designing species-specific probes (15–30 bases of oligonucleotides) that are arrayed into well plates and fixed on a nylon or nitrocellulose membrane. The probe hybridization with PCR amplified and labeled target DNA sequence can then be detected (Clark et al., 1999; Zhang et al., 2008). An oligo-DNA macroarray assay was established with digoxigenin-labeled RNA probes to identify the microbes in the phyllosphere of apple trees (He et al., 2012). For the identification of fungi, 40 bp of oligo-DNA sequences were selected from the fungal *rDNA-ITS* region and fungal pathogens that inflict scab (*Venturia inaequalis*), Alternaria blotch (*Alternaria mali*), and Marssonina blotch (*Diplocarpon mali*) were detected along with several non-phytopathogenic fungal and bacteria (He et al., 2012). DNA macroarray based reverse-blot hybridization was performed to target the *TUB2* gene from isolated DNA and amplified using Bt2a and Bt2b primers that were labelled with digoxygenin in PCR. This array endorsed the detection and discrimination of 15 different species belonging to the genera *Cadophora*, *Campylocarpon*, *Cylindrocarpon*, *Dactylonectria*, *Ilyonectria*, *Neonectria*, and *Phaeomoniella* including the black-foot pathogens *Campylocarpon fasciculare*, *C. pseudofasciculare*, *Dactylonectria macrodidyma, D. pauciseptata*, *Ilyonectria europaea*, *I. liriodendri*, and *I. robusta* (Agustí-Brisach and Armengol, 2013). Young vine decline (YVD) is a complex disease in grapevine that causes severe mortality in young vineyards (Úrbez-Torres et al., 2015). Sixty-one species including 34 YVD fungal pathogens were diagnosed by a DNA macroarray (Úrbez-Torres et al., 2015). Macroarray offers a reliable and effective method for pathogen diagnosis even if the sample contains multiple pathogens (Zhang et al., 2008). Finite life of filters, annotations of potential new genes showing low abundance of transcripts, and large volume of filters needed to be hybridized are major limitations of macroarray (Gammill and Lee, 2008). The other pitfalls of this array are lack of pathogen quantification and the impossibility to define whether the detected DNA is from a live organism (Úrbez-Torres et al., 2015).

### DNA or RNA Probe Based Assays

#### *In Situ* Hybridization

*In situ* hybridization (ISH) technique functions to detect mRNAs present in the fixed sample. Designing of an antisense ssRNA probe aimed to bind target mRNA (sequence of interest) is the main step of this assay. However, synthetic oligonucleotide probes and cDNA probes can also be used (Jensen, 2014). Typically, probes are labeled with the radioactive isotopes ^35^S, ^125^I, and ^32^P for probe labeling as they are very sensitive and easily quantified for detection. Nonisotopic probes can use biotin, digoxigenin, tyramide, alkaline phosphatase, or bromodeoxyuridine for probe labeling. Photography, autoradiography with X-ray film, liquid emulsion, and microscopic techniques can be used in signal detection (Corthell, 2014). The rust fungi *Puccinia horiana* isolate PA-11, *Uromyces transversalis* isolate CA-07, and *Phakopsora pachyrhizi* isolate Taiwan 72-1 infecting *Chrysanthemum* × *morifolium*, *Gladiolus* × *hortulanus*, and *Glycine max* respectively, were distinguished as rust pathogens in their respective paraffin fixed plant tissues using the ISH technique (Ellison et al., 2016). ISH enables maximum usage of tissue that is hard to obtain, but the main limitations of ISH are cost and hazardous nature of radioactive probes, and the difficulty in identification when the target has low concentrations of DNA and RNA (Jin and Lloyd, 1997).

#### Fluorescent *In Situ* Hybridization

Fluorescent *in situ* hybridization (FISH) is a comparatively recent and innovative technology in plant disease diagnostics. It combines the specificity in DNA sequences with the sensitivity of detection systems based on fluorochromes (Hijri, 2009; Cui et al., 2016). FISH assays include the detection of DNA or RNA sequences in cells or tissues using DNA or RNA probes, which are labeled directly or indirectly with fluorochromes (Shakoori, 2017). In normal FISH methods, fluorescently mono-labeled oligonucleotide probes are hybridized to the ribosomal RNA (rRNA) of microbial cells, and the stained cells are then visualized by wide field epifluorescence or confocal laser scanning microscopy (Lukumbuzya et al., 2019). Upon pathogen infection, pathogen-specific rRNA sequences will be present in plants. This specific information provided by RNA can be detected by FISH (Fang and Ramasamy, 2015). Southern blight in tomato is caused by a soil-borne pathogen, *Sclerotium rolfsii*. Soil smears in a DNA isolation with 0.06 pg µl^−1^of *S. rolfsii* was effectively detected by FISH technique that used an oligonucleotide probe labeled with cyanine dyes Cy3 and Cy5 (Milner et al., 2019). Reproducibility, sensitivity, specificity, accuracy, and speed are the best features of FISH (Bozorg-Ghalati et al., 2019). It also has potential to deliver information about resolution, morphology, and identification of main pathogens in mixed species specimens (Frickmann et al., 2017). False positive results with autofluorescence materials are a common pitfall that reduces specificity during the assay (Moter and Göbel, 2000).

### Next-Generation Sequencing

Next-generation sequencing (NGS) or high throughput sequencing (HTS) is a new approach for diagnostics. The development of NGS technologies has fueled innovative ways for detection and identification of phytopathogens (Chalupowicz et al., 2019). Isolation and fragmentation of DNA, library preparation, massive parallel sequencing, bioinformatics analysis, and variant/mutation annotation and interpretation are the major steps involved in DNA based NGS (Qin, 2019). Massively parallel signature sequencing, pyrosequencing, polony sequencing, and sequencing by oligonucleotide ligation detection (SOLID) are some commonly available advanced sequencing methods in HTS (Rajesh and Jaya, 2017). RNA-Sequencing (RNA-Seq) offers advanced coverage and greater resolution of the dynamic nature of the transcriptome. The Illumina HiSeq platform is the most universally functional NGS platform for RNA-Seq and has established the standard for NGS. The platform more recently released a desktop sequencer, MiSeq (Kukurba and Montgomery, 2015).

RNA-Seq based NGS can be used in the rapid identification of fungal plant pathogens inducing novel diseases. A whole-genome sequencing approach using Illumina MiSeq was established to detect the Sarcococca blight-causing novel fungal pathogen, *Calonectria pseudonaviculata* in ornamental plants. A 51.4 Mb genome of the two host isolates showed a unique single nucleotide polymorphism for the two isolates and identified both as *C. pseudonaviculata* (Malapi-Wight et al., 2016b). Datasets from population genomics built on NGS can be exploited to recuperate variations including single nucleotide polymorphisms (SNPs), insertions and deletions (INDELS), and structural variations (Potgieter et al., 2020). *Puccinia striiformis* f. sp. *tritici* (PST) is an emerging or re-emerging plant infecting fungus that causes wheat yellow (stripe) rust in wheat and triticale. Field pathogenomics was done using RNA-Seq based NGS of PST infested wheat leaves to gain insight into emergent pathogen populations. The results revealed that there was a dramatic shift in the PST population in the UK, likely due to a current introduction of a different set of emerging and exotic PST lineages (Hubbard et al., 2015). RNA and DNA based NGS approach was conducted to develop molecular diagnostics for the cucurbit downy mildew pathogen *Pseudoperonospora cubensis*. Comparative genomics using RNA-Seq of close relative species *P. humuli* identified seven specific regions in *P. cubensis* that allowed for the development of diagnostic markers (Withers et al., 2016).

*Monilinia fructicola*, a brown rot disease causing fungal pathogen, causes post- and pre-harvest damages in stone and pome fruits. A hybrid and hierarchical *de novo* association strategy was used to sequence the genome of *M. fructicola* Mfrc123 strain through an amalgamation of Illumina short read NGS and Pacific Biosciences (PacBio) long read third-generation sequencing platforms (Angelini et al., 2019). In another hybrid approach, the genome of the coffee rust fungus *Hemileia vastarix* was sequenced using PacBio RS II and Illumina HiSeq platforms and a total genome of 547 Mb of *H. vastatrix* race XXXIII was generated (Porto et al., 2019). The sequenced reference genomes can be used to study the genome biology and evolution with other species. The arrival of a novel pathogen is an instance where the target cannot be well-defined. Since NGS involves no prior knowledge of pathogen sequences, the whole genome of the causal organism may be sequenced without using specific primer pairs and PCR amplification (Hadidi et al., 2016; Malapi-Wight et al., 2016b). Among NGS approaches, advances in single-molecule sequencing technologies, “third-generation sequencing,” has advantages over second-generation sequencing techniques (Schadt et al., 2010). The major limitation in NGS is the time consumption incurred during assembly and analysis of large amounts of sequence data (Espindola et al., 2015). Next generation applications are often restricted by low RNA yield and/or integrity, RNA stability, and impurities with DNA, salts, or chemicals (Cortés-Maldonado et al., 2020). Also, bioinformatics and mycological expertise are necessary for NGS analysis, even if the data can be easily and quickly obtained, so that knowledge about fungal and bioinformatics analyses are mandatory to avoid any misinterpretation.

## Conclusion

Recent advances in molecular biological techniques have increased detection and diagnosis of novel, emerging, previously reported, and re-emerging fungal plant pathogens. Traditional and variant polymerase chain reaction (PCR) based assays, isothermal and post amplification tools, hybridization techniques, and next-generation sequencing (NGS) approaches are well-known for diagnostics in phytofungal disease detection. These molecular-based approaches have successfully identified and diagnosed symptomatic and asymptomatic diseases of culturable and unculturable fungal pathogens in sole and co-infections of agriculturally important field, horticultural, floricultural, ornamental, and forest plant species. Among various PCR centered assays, quantitative PCR has been extensively used in the quantification and differentiation of causal agents when the sample load is too insignificant to detect. Currently, loop-mediated amplification (LAMP) is showing success in the fungal disease detection arena and has facilitated identification of *Alternaria* spp., *Colletotrichum* spp., *Fusarium* spp., *Verticillium* spp., *Puccinia* spp., *Botrytis* spp., etc. that cause an array of devastating diseases in plants. NGS uses various platforms to sequence fungal genomes with no prior knowledge of the pathogen’s sequence, and can be used to identify novel and emerging pathogens.

The molecular methods covered in this review are accurate, effective, lab-oriented, and require sophisticated instruments to identify fungal plant pathogens. However, expertise in mycology and bioinformatics are essential to avoid any misinterpretation of the results obtained from the molecular biological analyses. Molecular techniques should become a point of care testing (POCT) by combining with other emergent technological advancements for fungal disease diagnosis. The challenges are with scientists to develop practical approaches for molecular diagnostics of crop diseases.

## Author Contributions

GH gathered literature and wrote the manuscript with input from KP. KP conceived the idea and provided guidance throughout and involved in critical revisions. All authors contributed to the article and approved the submitted version.

## Conflict of Interest

The authors declare that the research was conducted in the absence of any commercial or financial relationships that could be construed as a potential conflict of interest.
